# Supplementary material for: Sense of coherence and risk of breast cancer
Source: eLife. 2020 Nov 23;9:e61469. doi: 10.7554/eLife.61469 (PMC7717898; doi:10.7554/eLife.61469)
Supplement: Supplementary file 1. — The following questions were asked to investigate how the participants experienced these situations. A 7-point Likert-type scale is used to answer each question, with one corresponding to ‘very seldom or never’, and seven corresponding to ‘very often’. To simplify the structure, the answer of item 4 to 9, 11 to 13 have been reversed on the basis of the original SoC-13 questionnaire. [file elife-61469-supp1.pdf]

SoC-13 questionnaire of sense of coherence used in the Karolinska Mammography Project for Risk Prediction of Breast Cancer (Karma) cohort

The following questions were asked to investigate how the participants experienced these situations. A 7-point Likert-type scale is used to answer each question, with 1 corresponding to “very seldom or never”, and 7 corresponding to “very often”. To simplify the structure, the answer of item 4 to 9, 11 to 13 have been reversed on the basis of the original SoC-13 questionnaire.

Number	Question	Dimension
1	Do you have a sense that you do not care what is going on around you?	Meaningfulness
2	Have you been surprised by the behavior of people you thought you knew?	Comprehensibility
3	Has it happened that people you trusted have let you down?	Manageability
4	Do you think your life until now have a complete lack of goals?	Meaningfulness
5	Do you feel that you are treated unfair?	Manageability
6	Do you have a feeling of being in an unfamiliar situation without knowing what to do?	Comprehensibility
7	Are your daily activities a source to great joy and satisfaction?	Meaningfulness
8	Do you have disorganized emotions, thoughts and ideas shifting from one to another?	Comprehensibility
9	Do you have feelings that you would rather not acknowledge?	Comprehensibility
10	People, even with character, feel like sad losers in special situations. How often did you feel like this?	Manageability
11	When something happened, how often did you over or underestimate its importance?	Comprehensibility
12	How often have do you feel that there is little or no meaning with your daily life?	Meaningfulness
13	How often do you feel uncertain to manage to control yourself?	Manageability
